# Characterization of the Effects of a Humanin Fragment Peptide (HNF_14_) in Age-Related Macular Degeneration

**DOI:** 10.3390/jcm15051686

**Published:** 2026-02-24

**Authors:** Sonali Nashine, M. Cristina Kenney

**Affiliations:** Department of Ophthalmology, Gavin Herbert Eye Institute, University of California Irvine, Irvine, CA 92697, USA

**Keywords:** age-related macular degeneration, AMD, Humanin, Humanin G, HNF_14_, peptide

## Abstract

**Background:** Age-related macular degeneration (AMD) is a leading cause of vision loss and is strongly associated with mitochondrial dysfunction in retinal pigment epithelial cells. Mitochondrial-derived peptides, including Humanin and its analogs, have demonstrated cytoprotective effects in AMD-related cellular models. However, the effects of shorter Humanin-derived fragments in disease-specific mitochondrial models remain incompletely characterized. **Methods**: Transmitochondrial retinal pigment epithelial cybrid cell lines containing mitochondria from AMD patients or age-matched normal donors were treated with HNF_14_, a 14-amino acid Humanin fragment peptide. Cellular metabolic activity, cytotoxicity, oxidative stress, apoptotic signaling, inflammatory markers, angiogenic factor expression, and amyloid-β_1–42_-induced apoptosis were evaluated using biochemical assays, protein analyses, and live-cell imaging approaches. **Results**: HNF_14_ treatment was associated with improved metabolic activity and reduced cytotoxicity in AMD cybrids, with minimal effects in normal cybrids. HNF_14_ significantly reduced intracellular and mitochondrial oxidative stress, suppressed apoptotic and inflammatory markers, and decreased VEGF-A protein expression in AMD cybrids. In addition, HNF_14_ attenuated amyloid-β_1–42_-induced apoptotic signaling in AMD cybrids. These effects were selective for cybrids containing AMD-derived mitochondria. **Conclusions**: This study demonstrates that HNF_14_ mitigates mitochondrial and cellular stress responses in AMD transmitochondrial cybrid cells. The findings indicate that a short Humanin-derived fragment retains cytoprotective activity in a disease-specific mitochondrial context and support further investigation of mitochondrial-derived peptides as modulators of mitochondrial dysfunction relevant to AMD pathophysiology.

## 1. Introduction

Age-related macular degeneration (AMD) is a leading cause of irreversible central vision loss in older adults and represents a major clinical challenge worldwide [1,2,3,4]. The disease manifests as non-neovascular or neovascular AMD, both of which ultimately involve degeneration of the retinal pigment epithelium (RPE) and subsequent photoreceptor dysfunction [2,3]. While anti-vascular endothelial growth factor (VEGF) therapy has significantly improved outcomes for neovascular AMD, there are limited disease-modifying treatments for atrophic AMD, underscoring the need to identify upstream therapeutic targets that address fundamental cellular stress mechanisms [3].

Mitochondrial dysfunction is now recognized as a central contributor to AMD pathogenesis and oxidative stress [5]. RPE cells are among the most metabolically active cells in the body and depend heavily on mitochondrial oxidative phosphorylation to sustain retinal homeostasis. Studies using patient-derived tissues and cellular models have demonstrated that AMD mitochondria exhibit impaired bioenergetics, increased reactive oxygen species production, mitochondrial DNA damage, and heightened susceptibility to apoptosis [6,7,8]. These mitochondrial abnormalities are thought to occur early in disease progression and may drive downstream inflammatory, angiogenic, and degenerative processes, positioning mitochondria as a promising therapeutic target [9].

Mitochondrial-derived peptides (MDPs) are small bioactive peptides encoded within the mitochondrial genome that play key roles in cellular stress resistance and survival. Humanin, the first discovered MDP, is encoded within the *MT-RNR2* gene and has been extensively characterized for its cytoprotective effects across multiple disease models. Humanin and its analogs protect against oxidative stress, apoptosis, inflammation, and amyloid-β-induced toxicity [10,11,12,13,14]. Importantly, circulating Humanin levels decline with age and are significantly reduced in patients with AMD, suggesting a loss of endogenous mitochondrial protection in this disease context [8,15,16,17].

Previous work using transmitochondrial RPE cybrid models has demonstrated that exogenous administration of Humanin G (HNG), a potent single-amino acid variant of Humanin, rescues AMD cybrids from mitochondrial and cellular damage [9,18]. HNG treatment reduces apoptosis, oxidative stress, inflammation, and angiogenic signaling while improving mitochondrial integrity and cell survival in AMD cybrids [9,10,11,19,20]. These studies established mitochondrial-derived peptides as promising therapeutic candidates for AMD and validated the cybrid model as a disease-relevant platform for evaluating mitochondria-targeted interventions.

Despite these advances, the therapeutic potential of shorter Humanin-derived fragments remains incompletely explored in AMD. Shorter peptides may retain essential bioactivity while offering advantages related to synthesis, stability, and translational development. Humanin Fragment 14 (HNF_14_) is a 14-amino acid peptide (molecular weight 1653.7 Da) derived from the Humanin sequence, with the amino acid sequence LLLTSEIDLPVKRR (N→C). HNF_14_ represents a distinct class of Humanin-based peptides that has not previously been evaluated in disease-specific mitochondrial models of AMD.

Transmitochondrial cybrid models provide a powerful experimental system in which RPE cells share an identical nuclear background but harbor mitochondria derived from either AMD patients or age-matched normal donors. This approach isolates the contribution of mitochondrial DNA to disease phenotypes and has consistently demonstrated that AMD cybrids exhibit increased oxidative stress, apoptotic signaling, inflammatory activation, and dysregulated angiogenic factor expression compared to normal cybrids [9,11,21].

In the present study, we investigated the effects of HNF_14_ on mitochondrial dysfunction and cell survival pathways in AMD cybrids. We hypothesized that HNF_14_ would selectively mitigate oxidative stress, cytotoxicity, apoptotic signaling, and angiogenic factor expression in AMD cybrids while exerting minimal effects in normal cybrids. By evaluating functional, molecular, and live-cell imaging endpoints, this study aims to determine whether a short Humanin-derived fragment can preserve mitochondrial and cellular health in a disease-specific AMD context and thereby support mitochondrial targeting as a therapeutic strategy for AMD.

## 2. Materials and Methods

### 2.1. Human Subject Research

Research involving human participants was approved by the Institutional review board of the University of California, Irvine (#2003-3131). Written informed consent was obtained from all subjects and clinical investigations were conducted according to the tenets of Declaration of Helsinki for research involving human subjects.

### 2.2. Transmitochondrial Cybrid Cell Lines

Transmitochondrial retinal pigment epithelial cybrid cell lines were created by fusing mitochondrial DNA-deficient ARPE-19 (Rho0) cell line with platelets isolated from either AMD patients or age-matched normal subjects. Peripheral blood was collected via venipuncture in tubes containing 10 mM EDTA, and blood platelets were isolated by a series of centrifugation steps. To create the ARPE-19 Rho0 cells, ARPE-19 cells were serially passaged into medium containing ethidium bromide. Cybrids were created by polyethylene glycol fusion of platelets with Rho0 ARPE-19 cells in medium containing uridine. After growing the cybrid cells in uridine-containing medium for 2 weeks, the medium was replaced with regular culture medium. All transmitochondrial cybrids were grown in DMEM/Ham’s F12 1:1 (Invitrogen-Gibco, Gaithersburg, MD, USA) cell culture medium containing 24 mM sodium bicarbonate, 10% dialyzed fetal bovine serum, and 1.0 mM sodium pyruvate. Cells were maintained at 37 °C in a humidified incubator containing 5% CO_2_. This approach yielded cybrid lines that shared an identical nuclear background but differed in mitochondrial DNA origin. Each cybrid line represented a single individual donor. Passage 5 cybrids were used for all experiments. Age-matched normal cybrids served as controls.

### 2.3. HNF_14_ Peptide Treatment

HNF_14_ is a 14-amino acid Humanin-derived fragment peptide with ≥96.0% purity, custom synthesized by AnaSpec, Inc. (Fremont, CA, USA). The peptide has a molecular weight of 1653.7 Da and the amino acid sequence LLLTSEIDLPVKRR (N→C). Lyophilized HNF_14_ peptide was reconstituted in sterile water to prepare a 1 mM stock solution, which was subsequently diluted in cell culture medium to obtain a 3.2 µM working solution. The 3.2 µM concentration of HNF_14_ was selected based on prior studies using Humanin G (HNG) in AMD transmitochondrial cybrids. In addition, exploratory concentration testing with HNF_14_, including 1 µM, indicated that 3.2 µM produced the most robust protective effects. Accordingly, this concentration was used for all experiments. Cybrid cells were treated with HNF_14_ at a final concentration of 3.2 µM for 48 h. All downstream assays were performed following the 48-h treatment period unless otherwise specified.

### 2.4. MTT Assay

The MTT assay is a colorimetric test that utilizes the phenomenon of reduction of tetrazolium salts to measure cell viability. Healthy actively metabolizing cells contain NAD(P)H-dependent cellular oxidoreductase enzymes that reflect the number of viable cells present. These enzymes reduce the water-soluble yellow tetrazolium dye MTT [3-(4,5-dimethylthiazol-2-yl)-2,5-diphenyltetrazolium bromide] to its insoluble formazan, which has a purple color. The formazan crystals are then solubilized by adding dimethyl sulfoxide (DMSO) and the colorimetric signal, which is proportional to the number of viable cells, is determined by measurement of optical density at 570 nm. MTT assays were performed in the dark since the MTT reagent is sensitive to light. AMD and normal cybrids plated in 96-well tissue culture plates were treated with MTT solution (Catalog number: 30006, Biotium, Fremont, CA, USA) at the 48 h time point. MTT-treated cells were incubated at 37 °C for 1 h, followed by addition of DMSO. Absorbance was measured on a spectrophotometer at 570 nm and background absorbance measured at 630 nm. Background absorbance was subtracted from signal absorbance to obtain normalized absorbance values. The colorimetric signal obtained was proportional to the cell number. Data were normalized to untreated normal controls.

### 2.5. LDH Cytotoxicity Assay

Cellular cytotoxicity was assessed by measuring lactate dehydrogenase (LDH) release into the culture medium using the CyQUANT™ LDH Cytotoxicity Assay Kit (Invitrogen/Thermo Fisher Scientific, Waltham, MA, USA; Catalog number: C20300) according to the manufacturer’s instructions. Cybrid cells were plated at a uniform density of 10,000 cells per well in 96-well plates to ensure consistent baseline conditions across wells. LDH release was assessed after 48 h of treatment and normalized to untreated controls. Briefly, AMD and normal transmitochondrial cybrid cells were treated with HNF_14_ for 48 h, after which cell culture supernatants were collected and transferred to a 96-well flat-bottom plate. An equal volume of LDH reaction mixture was added to each well and incubated at room temperature for 30 min protected from light. The reaction was stopped by addition of stop solution, and absorbance was measured at 490 nm with 680 nm as a reference wavelength. LDH activity was calculated by subtracting background absorbance at 680 nm from the 490 nm signal. Data were normalized to untreated normal cybrids.

### 2.6. Intracellular ROS Assay

Intracellular reactive oxygen species (ROS) levels were measured using 2′,7′-dichlorodihydrofluorescein diacetate (H_2_DCFDA) (Sigma-Aldrich, St. Louis, MO, USA; Catalog number: 287810), a cell-permeant fluorogenic probe. H_2_DCFDA is a non-fluorescent, chemically reduced form of fluorescein, that following cleavage of its acetate groups by intracellular esterases and subsequent oxidation, is converted to the highly fluorescent 2′,7′-dichlorofluorescein (DCF). Cybrid cells were incubated with H_2_DCFDA as indicated, and fluorescence was measured using excitation and emission wavelengths of 492 nm and 520 nm, respectively. Fluorescence values were background-corrected using cell-free blanks containing H_2_DCFDA reagent alone, and signals were normalized to untreated controls. Fluorescence intensity was proportional to intracellular ROS levels and was normalized to untreated controls.

### 2.7. MitoSOX Assay

Mitochondrial superoxide production was assessed using MitoSOX™ Red mitochondrial superoxide indicator (Invitrogen/Thermo Fisher Scientific, Waltham, MA, USA; Catalog number: M36008), a live-cell permeant fluorogenic dye that selectively detects mitochondrial superoxide. A 5 mM MitoSOX Red stock solution was diluted in Hank’s Balanced Salt Solution (HBSS) to prepare a 5 µM working solution. Cells were incubated with 5 µM MitoSOX Red for 10 min at 37 °C, protected from light. Following incubation, cells were washed with HBSS, and MitoSOX fluorescence was quantified at excitation/emission wavelengths of 510/580 nm, providing a quantitative measure of mitochondrial superoxide levels. Fluorescence intensity was quantified and normalized to untreated controls.

### 2.8. Protein Extraction and Quantification

Normal and AMD transmitochondrial cybrid cells were plated in six-well plates and treated with HNF_14_. Cells were lysed using RIPA buffer (Thermo Fisher Scientific, Waltham, MA, USA; Catalog number: 89900) supplemented with protease inhibitors according to the manufacturer’s instructions. Cell lysates were clarified by centrifugation at 13,000× *g* for 10 min at 4 °C (using Prism R centrifuge, Labnet International, Woodbridge, NJ, USA), and the resulting supernatants were collected for protein analysis. Total protein concentrations were determined using the DC™ Protein Assay Kit (Bio-Rad Laboratories, Richmond, CA, USA) according to the manufacturer’s protocol. Equal amounts of protein were used for subsequent ELISA analyses.

### 2.9. VEGF-A ELISA

VEGF-A protein levels were quantified using a Human VEGF-A ELISA Kit (Invitrogen/Thermo Fisher Scientific, Waltham, MA, USA; Catalog number: BMS277-2) according to the manufacturer’s instructions. Briefly, AMD and normal transmitochondrial cybrid cells were treated with HNF_14_ for 48 h, after which cell culture supernatants were collected, clarified by centrifugation (using Prism R centrifuge, Labnet International, Woodbridge, NJ, USA), and stored at −20 °C until analysis. Samples and standards were assayed in duplicate on microplates pre-coated with an anti-human VEGF-A capture antibody. Following incubation with a biotin-conjugated anti-VEGF-A detection antibody and streptavidin-HRP, color development was achieved using tetramethylbenzidine substrate and the reaction was stopped with phosphoric acid. Absorbance was measured at 450 nm with 620 nm as a reference wavelength using a microplate reader. VEGF-A concentrations were calculated from a standard curve generated using recombinant human VEGF-A standards. Data were normalized to untreated normal cybrids.

### 2.10. Annexin V ELISA

Annexin V protein levels were quantified using a commercially available Human Annexin V ELISA Kit (Invitrogen/Thermo Fisher Scientific, Waltham, MA, USA; Catalog number: BMS252) according to the manufacturer’s instructions. Although Annexin V is not classically secreted, extracellular Annexin V detected in the culture medium reflects apoptotic membrane remodeling and release from stressed or dying cells. Accordingly, Annexin V levels in the supernatant were used as a quantitative indicator of apoptotic burden. Briefly, AMD and normal transmitochondrial cybrid cells were treated with HNF_14_ for 48 h, after which cell culture supernatants were collected, clarified by centrifugation, and stored at −20 °C until analysis. Samples and standards were assayed in microplates pre-coated with an anti-human Annexin V capture antibody. Following incubation with a biotin-conjugated anti-Annexin V detection antibody and streptavidin–HRP, color development was achieved using tetramethylbenzidine substrate and the reaction was stopped with phosphoric acid. Absorbance was measured at 450 nm. Annexin V concentrations were calculated from a standard curve generated using recombinant human Annexin V standards. Data were normalized to untreated normal cybrids.

### 2.11. Caspase-3 (Active/Cleaved) ELISA

Active (cleaved) caspase-3 protein levels were quantified using a Human Caspase-3 (Active) ELISA Kit (Invitrogen™/Thermo Fisher Scientific, Waltham, MA, USA; Catalog number: KHO1091) according to the manufacturer’s instructions. This solid-phase sandwich ELISA specifically detects human caspase-3 cleaved at Asp175/Ser176. Following HNF_14_ treatment, AMD and normal cybrid cells were harvested, washed with cold phosphate-buffered saline (PBS), and lysed in Cell Extraction Buffer supplemented with protease inhibitors. Lysates were clarified by centrifugation at 13,000× *g* for 10 min at 4 °C (using Prism R centrifuge, Labnet International, Woodbridge, NJ, USA), and supernatants were collected for analysis. Equal amounts of clarified cell lysates were diluted in Standard Diluent Buffer and added to antibody-coated 96-well plates along with recombinant active caspase-3 standards. Plates were incubated at room temperature, washed, and sequentially incubated with caspase-3 (active) detection antibody followed by HRP-conjugated secondary antibody. Color development was achieved using tetramethylbenzidine (TMB) substrate and the reaction was stopped with stop solution. Absorbance was measured at 450 nm. Active caspase-3 concentrations were calculated from a standard curve generated using recombinant human caspase-3 standards. Data were normalized to untreated normal cybrids.

### 2.12. Amyloid-β_1–42_ Peptide Treatment

Lyophilized amyloid-β_1–42_ (Aβ_1–42_; active form) peptide (AnaSpec, Inc., Fremont, CA, USA; Catalog number: AS-20276) and amyloid-β_42–1_ (Aβ_42–1_; scrambled inactive control) peptide (AnaSpec, Inc.; Fremont, CA, USA; Catalog number: 27276) were reconstituted in 1% ammonium hydroxide to prepare stock solutions, which were subsequently diluted in 1× phosphate-buffered saline (PBS) to obtain a 20 µM working solution. Cybrid cells were treated with 20 µM amyloid-β peptides as indicated. To assess apoptotic responses under amyloid-associated stress, AMD cybrids were exposed to Aβ_1–42_ in the presence or absence of HNF_14_.

### 2.13. IncuCyte Live-Cell Imaging for Caspase-3/7 Activity

Live-cell apoptosis was assessed using the IncuCyte^®^ Caspase-3/7 Green Apoptosis Reagent (Catalog number: 4440, IncuCyte, Sartorius, Ann Arbor, MI, USA), which couples the activated caspase-3/7 recognition motif (DEVD) to a DNA-intercalating dye, enabling real-time detection of caspase-3/7-mediated apoptosis in live cells. The reagent is an inert, non-fluorescent substrate that freely crosses the cell membrane and is cleaved by activated caspase-3/7, resulting in fluorescent labeling of nuclear DNA. Cells were seeded in 96-well plates and treated with HNF_14_ as indicated. Cells were then incubated with IncuCyte^®^ Caspase-3/7 Green reagent (1:1000; final concentration 5 µM) together with IncuCyte^®^ NucLight Rapid Red nuclear dye (Catalog number: 4717, IncuCyte; 1:500 dilution), a live-cell permeable DNA stain used to label total nuclei. Plates were placed into the IncuCyte^®^ Live-Cell Analysis System and allowed to equilibrate at 37 °C for 30 min prior to imaging. Fluorescent images were acquired at ~2-h intervals and fluorescent objects were quantified using the IncuCyte^®^ integrated analysis software (IncuCyte, Sartorius, Ann Arbor, MI, USA). Caspase-3/7 activity was expressed as the ratio of caspase-3/7-positive (green) nuclear objects to total NucLight Rapid Red-labeled nuclei, thereby normalizing apoptotic signal to cell number. NucLight Rapid Red was used exclusively for normalization and was not analyzed as an independent experimental endpoint.

### 2.14. Statistical Analysis

Experiments were performed using multiple independent transmitochondrial cybrid lines per group. All n values represent independent biological replicates, defined as separate transmitochondrial cybrid cell lines derived from individual donors. Technical replicates were not considered independent n values. Data are presented as mean ± standard deviation (SD). Statistical differences between groups were analyzed using one-way analysis of variance (ANOVA) followed by Tukey’s post hoc test. All statistical analyses were performed using GraphPad Prism version 5.0 (GraphPad Software, San Diego, CA, USA). P-values were calculated using GraphPad Prism, and significance annotations shown in figures correspond to results obtained from the statistical analysis. A *p* value < 0.05 was considered statistically significant.

## 3. Results

### 3.1. Effects of HNF_14_ on Cellular Metabolic Activity in AMD Cybrids (Figure 1)

**Figure 1:**
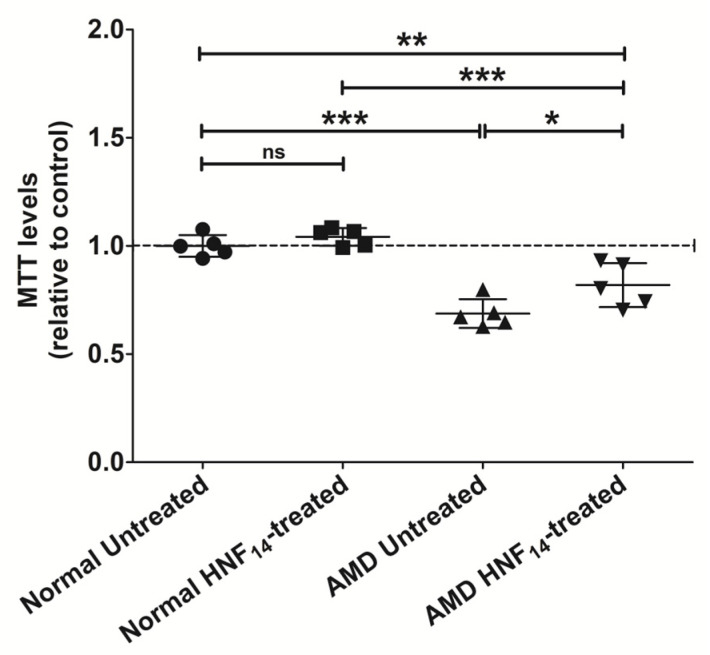
**Effects of HNF_14_ on cellular metabolic activity in AMD cybrids.** Cellular metabolic activity was assessed using an MTT assay in normal and age-related macular degeneration (AMD) transmitochondrial cybrid cells treated with HNF_14_ (3.2 µM) for 48 h. Individual data points represent biological replicates, and horizontal bars indicate the mean ± SD (*n* = 5 biological replicates). Data were normalized to untreated normal cybrids. Statistical significance is indicated as * *p* < 0.05, ** *p* < 0.01, *** *p* < 0.001; ns, not significant.

To evaluate the effects of HNF_14_ on cellular metabolic activity, AMD and normal transmitochondrial cybrid cells were treated with HNF_14_ (3.2 µM) for 48 h and analyzed using an MTT assay (Figure 1). Data are presented as mean ± SD (*n* = 5 biological replicates) and normalized to untreated normal cybrids. Under basal conditions, AMD cybrids demonstrated significantly lower metabolic activity compared with normal cybrids (NL UN: 1.000 ± 0.050; AMD UN: 0.687 ± 0.066; *p* < 0.001). Treatment with HNF_14_ significantly increased metabolic activity in AMD cybrids relative to untreated AMD controls (AMD UN: 0.687 ± 0.066; AMD HNF_14_: 0.819 ± 0.101; *p* < 0.05). In contrast, HNF_14_ treatment did not produce a significant change in metabolic activity in normal cybrids (ns), suggesting a disease-specific response.

### 3.2. Effects of HNF_14_ on Cytotoxicity in AMD Cybrids (Figure 2)

**Figure 2:**
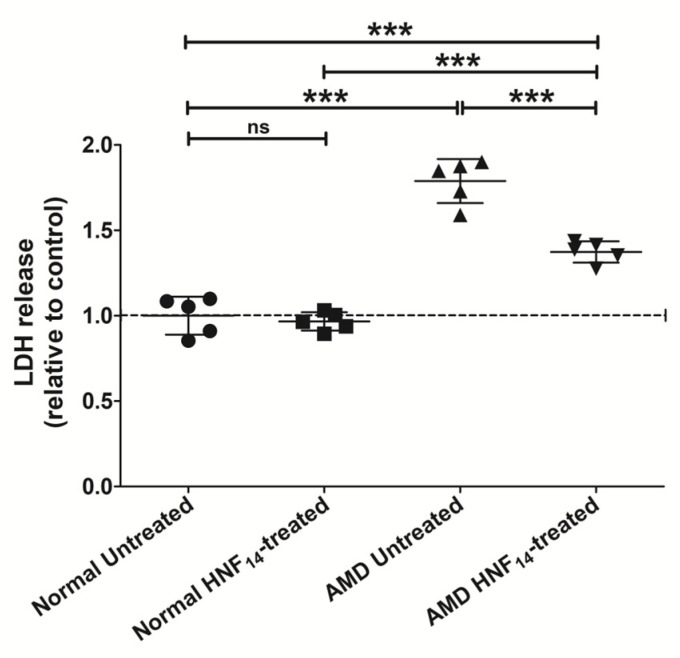
**Effects of HNF_14_ on cytotoxicity in AMD cybrids.** Cytotoxicity was evaluated by measuring lactate dehydrogenase (LDH) release into the culture medium following 48 h of HNF_14_ treatment. Individual data points represent biological replicates, with mean ± SD shown (*n* = 5 biological replicates). Values were normalized to untreated normal cybrids. Statistical significance is indicated as *** *p* < 0.001; ns, not significant.

Cytotoxicity was evaluated by measuring lactate dehydrogenase (LDH) release into the culture medium following 48 h of HNF_14_ treatment (Figure 2). Data are expressed as mean ± SD (*n* = 5 biological replicates) and normalized to untreated normal cybrids. Under basal conditions, AMD cybrids exhibited significantly elevated LDH levels compared with normal cybrids (NL UN: 1.000 ± 0.111; AMD UN: 1.789 ± 0.129; *p* < 0.001), suggesting increased cytotoxicity. Treatment with HNF_14_ significantly reduced LDH release in AMD cybrids relative to untreated AMD controls (AMD UN: 1.789 ± 0.129; AMD HNF_14_: 1.374 ± 0.063; *p* < 0.001). In contrast, HNF_14_ treatment did not significantly alter LDH levels in normal cybrids compared with untreated normal controls (ns).

### 3.3. Effect of HNF_14_ on Intracellular Oxidative Stress in AMD Cybrids (Figure 3)

**Figure 3:**
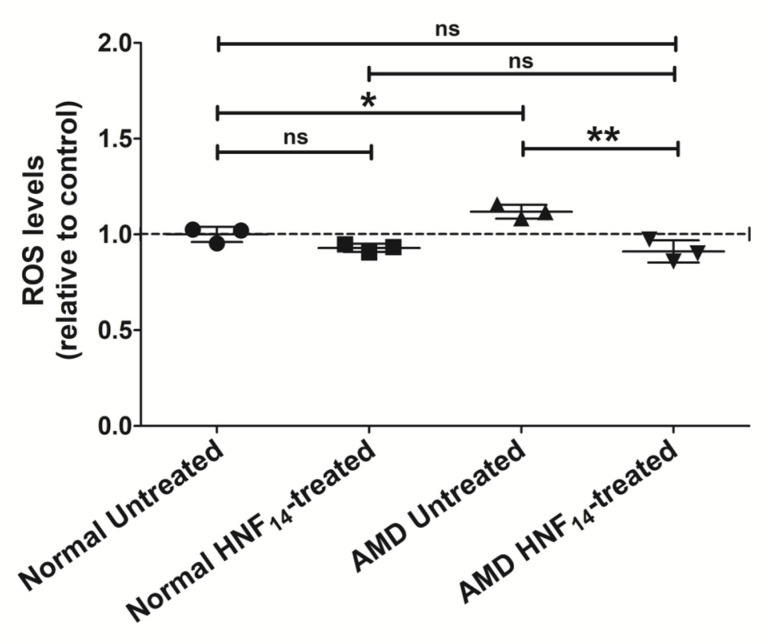
**Effects of HNF_14_ on intracellular oxidative stress in AMD cybrids.** Intracellular reactive oxygen species (ROS) levels were measured using the H_2_DCFDA assay following HNF_14_ treatment. Fluorescence was quantified using a fluorescence plate reader and normalized to untreated normal cybrids after background subtraction from cell-free blanks. Individual data points represent biological replicates, with mean ± SD shown (*n* = 3 biological replicates). Statistical significance is indicated as * *p* < 0.05, ** *p* < 0.01; ns, not significant.

Intracellular reactive oxygen species (ROS) levels measured using an H_2_DCFDA assay were significantly higher in AMD cybrids compared with normal cybrids (NL UN: 1.000 ± 0.040; AMD UN: 1.119 ± 0.036; *p* < 0.05; Figure 3). Data are expressed as mean ± SD (*n* = 3 biological replicates) and normalized to untreated normal cybrids. Treatment with HNF_14_ significantly reduced intracellular ROS levels in AMD cybrids relative to untreated AMD controls (AMD UN: 1.119 ± 0.036; AMD HNF_14_: 0.912 ± 0.058; *p* < 0.01). In contrast, HNF_14_ treatment did not significantly alter intracellular ROS levels in normal cybrids (ns).

### 3.4. Effect of HNF_14_ on Mitochondrial Superoxide Production in AMD Cybrids (Figure 4)

**Figure 4:**
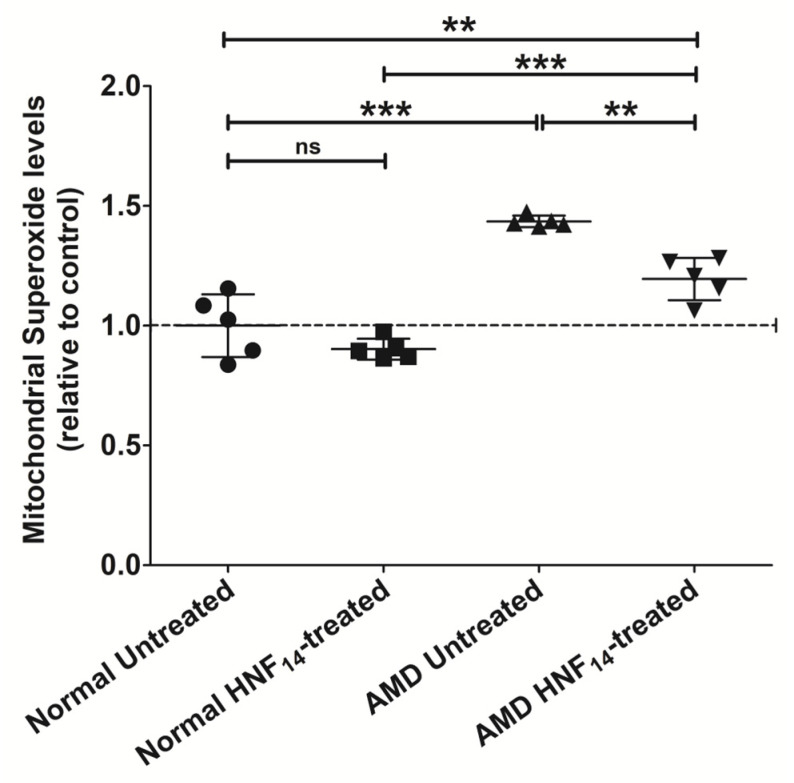
**Effects of HNF_14_ on mitochondrial superoxide production in AMD cybrids.** Mitochondrial superoxide levels were assessed using a MitoSOX™ Red-based assay following HNF_14_ treatment. Fluorescence was measured using a fluorescence plate reader and normalized to untreated normal cybrids after background subtraction. Individual data points represent biological replicates, with mean ± SD shown (*n* = 5 biological replicates). Statistical significance is indicated as ** *p* < 0.01, *** *p* < 0.001; ns, not significant.

Mitochondrial superoxide production, measured using a MitoSOX-based assay, was significantly increased in AMD cybrids compared with normal cybrids (NL UN: 1.000 ± 0.131; AMD UN: 1.435 ± 0.024; *p* < 0.001; Figure 4). Data are expressed as mean ± SD (*n* = 5 biological replicates) and normalized to untreated normal cybrids. HNF_14_ treatment significantly reduced mitochondrial superoxide levels in AMD cybrids (AMD UN: 1.435 ± 0.024; AMD HNF_14_: 1.195 ± 0.089; *p* < 0.01), whereas no significant effect was observed in normal cybrids treated with HNF_14_ (ns).

### 3.5. Effect of HNF_14_ on VEGF-A Protein Levels in AMD Cybrids (Figure 5)

**Figure 5:**
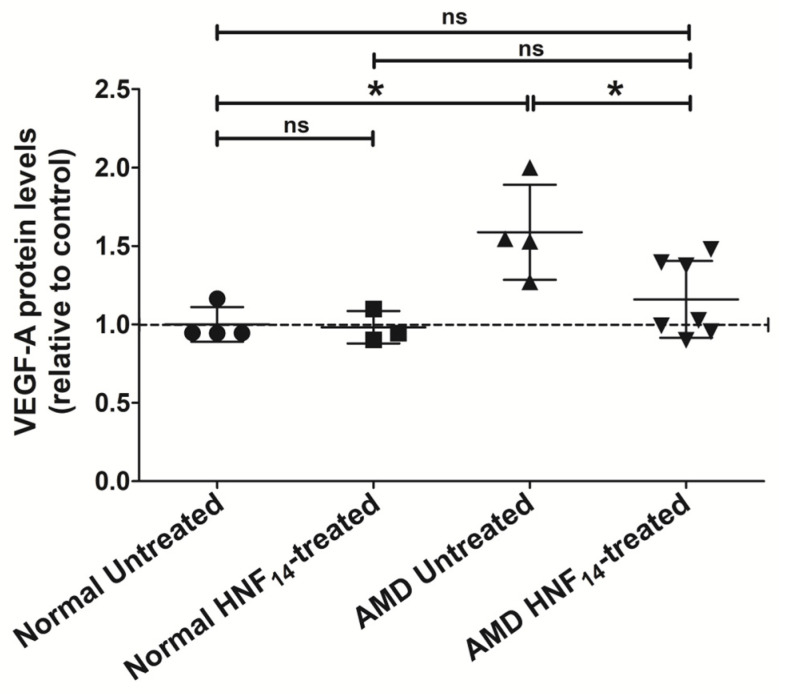
**Effects of HNF_14_ on VEGF-A protein levels in AMD cybrids.** VEGF-A protein levels were quantified by ELISA in normal and AMD cybrid cells following 48 h of HNF_14_ treatment. Individual data points represent biological replicates, and bars indicate mean ± SD (*n* = 3–7 biological replicates). Values were normalized to untreated normal cybrids. Statistical significance is indicated as * *p* < 0.05; ns, not significant.

VEGF-A protein levels, measured using an ELISA, were significantly higher in AMD cybrids compared with normal cybrids (NL UN: 1.000 ± 0.110; AMD UN: 1.587 ± 0.303; *p* < 0.05; Figure 5). Data are expressed as mean ± SD *(n* = 3–7 biological replicates) and normalized to untreated normal cybrids. Treatment with HNF_14_ significantly reduced VEGF-A protein levels in AMD cybrids relative to untreated AMD controls (AMD UN: 1.587 ± 0.303; AMD HNF_14_: 1.160 ± 0.245; *p* < 0.05). In contrast, HNF_14_ treatment did not significantly alter VEGF-A protein levels in normal cybrids compared with untreated normal controls (ns).

### 3.6. Effect of HNF_14_ on Annexin V Protein Levels in AMD Cybrids (Figure 6)

**Figure 6:**
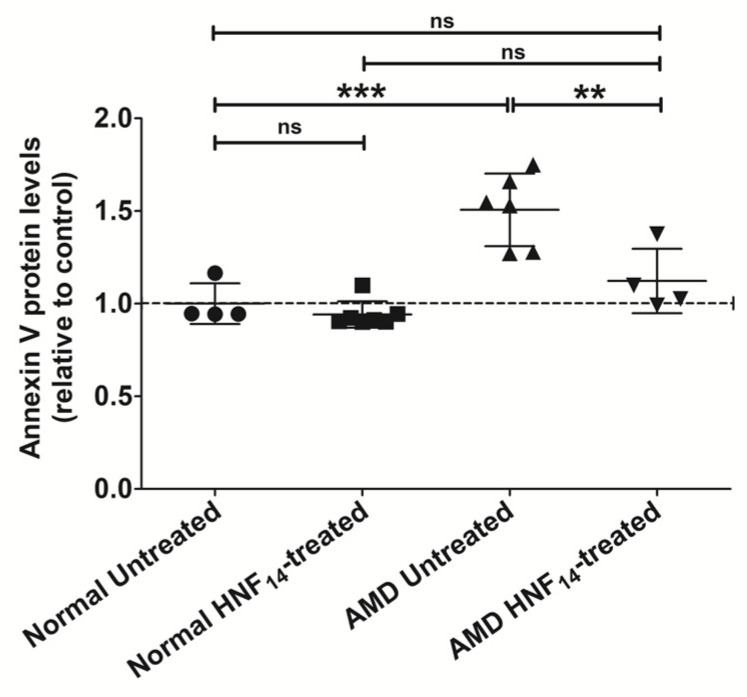
**Effects of HNF_14_ on Annexin V protein levels in AMD cybrids.** Early apoptotic signaling was assessed by measuring Annexin V protein levels in cell lysates using ELISA following 48 h of HNF_14_ treatment. Individual data points represent biological replicates, with mean ± SD shown (*n* = 4–7 biological replicates). Data were normalized to untreated normal cybrids. Statistical significance is indicated as ** *p* < 0.01, *** *p* < 0.001; ns, not significant.

Early apoptotic signaling was evaluated by measuring Annexin V protein levels using an ELISA following 48 h of HNF_14_ treatment (Figure 6). Data are expressed as mean ± SD and normalized to untreated normal cybrids (*n* = 4–7 biological replicates). Annexin V protein levels were significantly higher in untreated AMD cybrids compared with untreated normal cybrids (NL UN: 1.000 ± 0.110; AMD UN: 1.506 ± 0.196; *p* < 0.001). Treatment with HNF_14_ significantly reduced Annexin V protein levels in AMD cybrids relative to untreated AMD controls (AMD UN: 1.506 ± 0.196; AMD HNF_14_: 1.123 ± 0.174; *p* < 0.01). In contrast, HNF_14_ treatment did not significantly alter Annexin V protein levels in normal cybrids compared with untreated normal controls (ns).

### 3.7. Effect of HNF_14_ on Cleaved Caspase-3 (Active) Protein Levels in AMD Cybrids (Figure 7)

**Figure 7:**
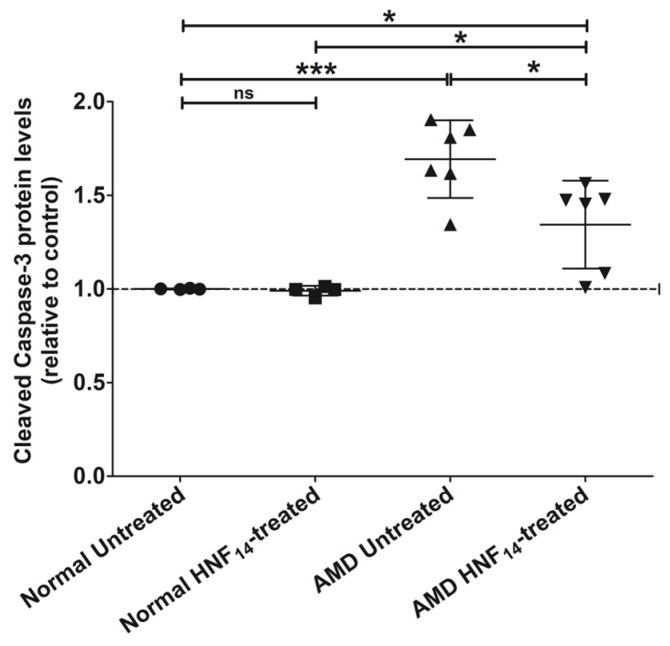
**Effects of HNF_14_ on cleaved caspase-3 protein levels in AMD cybrids**. Cleaved caspase-3 (active) protein levels were measured by ELISA following 48 h of HNF_14_ treatment. Individual data points represent biological replicates, with mean ± SD shown (*n* = 4–6 biological replicates). Values were normalized to untreated normal cybrids. Statistical significance is indicated as * *p* < 0.05, *** *p* < 0.001; ns, not significant.

Cleaved Caspase-3 protein levels were quantified by ELISA following 48 h of HNF_14_ treatment in AMD and normal cybrid cells (Figure 7). Data are expressed as mean ± SD and normalized to untreated normal cybrids (*n = 4–6 biological replicates*). Untreated AMD cybrids exhibited significantly higher cleaved caspase-3 protein levels compared with untreated normal cybrids (NL UN: 1.000 ± 0.003; AMD UN: 1.693 ± 0.207; *p* < 0.001). Treatment with HNF_14_ significantly reduced cleaved caspase-3 protein levels in AMD cybrids relative to untreated AMD controls (AMD UN: 1.693 ± 0.207; AMD HNF_14_: 1.343 ± 0.235; *p* < 0.05). However, cleaved caspase-3 levels in HNF_14_-treated AMD cybrids remained significantly higher than in both untreated and HNF_14_-treated normal cybrids (*p* < 0.05). In normal cybrids, HNF_14_ treatment did not significantly alter cleaved caspase-3 protein levels compared with untreated normal controls (ns).

### 3.8. Effect of HNF_14_ on Caspase-3/7 Activity in AMD Cybrids (Figure 8)

**Figure 8:**
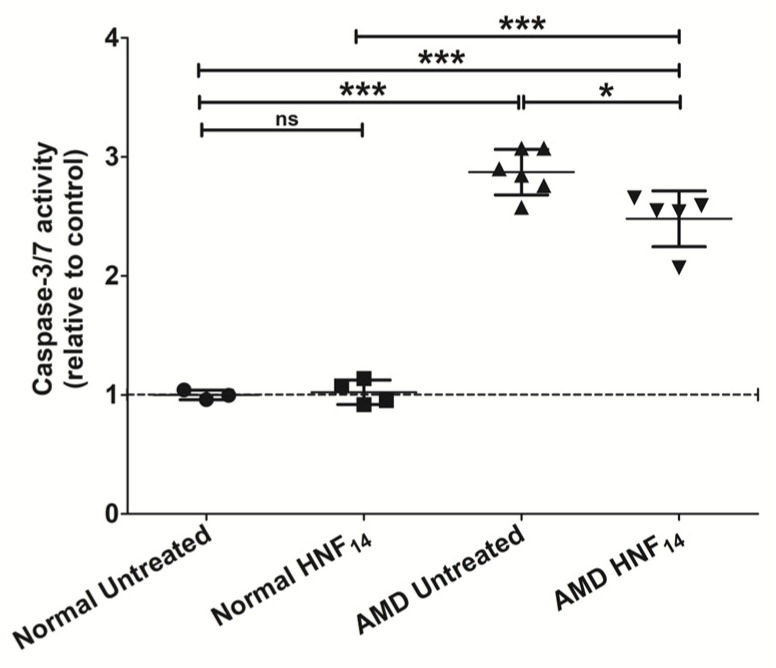
**Effects of HNF_14_ on caspase-3/7 activity in AMD cybrids assessed by IncuCyte live-cell imaging.** Caspase-3/7 activity was quantified using IncuCyte live-cell imaging and normalized to total nuclear count (NucLight™ Rapid Red). Individual data points represent biological replicates, with mean ± SD shown (*n* = 3–6 biological replicates). Data were normalized to untreated normal cybrids. Statistical significance is indicated as * *p* < 0.05, *** *p* < 0.001; ns, not significant.

Apoptosis was assessed using IncuCyte live-cell imaging by quantifying caspase-3/7 activity relative to nuclear count following HNF_14_ treatment (Figure 8). Data are expressed as mean ± SD and normalized to untreated normal cybrids (n = 3–6 biological replicates). Untreated AMD cybrids exhibited significantly higher caspase-3/7 activity compared with untreated normal cybrids (NL UN: 1.000 ± 0.040; AMD UN: 2.872 ± 0.191; *p* < 0.001). Treatment with HNF_14_ significantly reduced caspase-3/7 activity in AMD cybrids relative to untreated AMD controls (AMD UN: 2.872 ± 0.191; AMD HNF_14_: 2.480 ± 0.235; *p* < 0.05). However, caspase-3/7 activity in HNF_14_-treated AMD cybrids remained higher than that observed in untreated and HNF14-treated normal (*p* < 0.001). In normal cybrids, HNF_14_ treatment did not significantly alter caspase-3/7 activity compared with untreated normal controls (ns).

### 3.9. Effect of HNF_14_ on Amyloid-β_1–42_-Induced Caspase-3/7-Mediated Apoptosis in AMD Cybrids (Figure 9)

**Figure 9:**
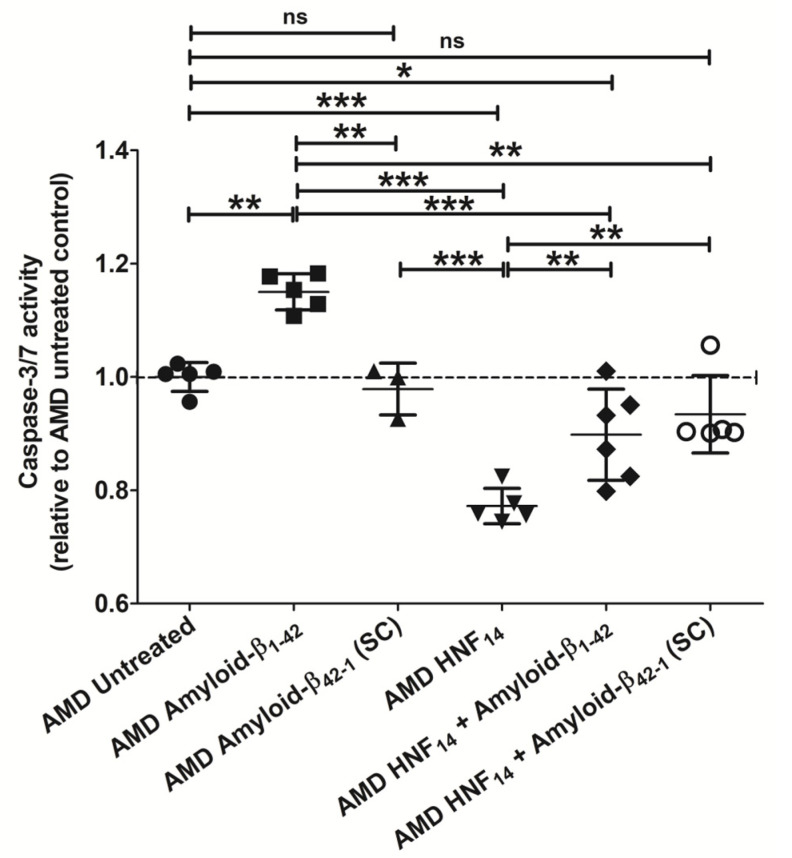
**Effects of HNF_14_ on amyloid-β_1–42_-induced caspase-3/7-mediated apoptosis in AMD cybrids.** Caspase-3/7 activity was assessed in AMD cybrids treated with amyloid-β_1–42_, scrambled control peptide (Aβ_42–1_), HNF_14_, or their combinations. Caspase-3/7 activity was normalized to nuclear count and expressed relative to untreated AMD controls. Individual data points represent biological replicates, with mean ± SD shown (*n* = 3–6 biological replicates). Statistical significance is indicated as * *p* < 0.05, ** *p* < 0.01, *** *p* < 0.001; ns, not significant.

Apoptosis was assessed by IncuCyte live-cell imaging and normalized to nuclear count following treatment with amyloid-β_1–42_ (Aβ_1–42_), HNF_14_, scrambled control peptide (Aβ_42–1_-SC), or their combinations (Figure 9). Data are expressed as mean ± SD and normalized to untreated AMD cybrids *(n* = 3–6 biological replicates). Treatment with Aβ_1–42_ significantly increased caspase-3/7 activity in AMD cybrids compared with untreated AMD controls (AMD UN: 1.000 ± 0.026; AMD Amyloid-β_1–42_: 1.150 ± 0.032; *p* < 0.01). HNF_14_ treatment alone significantly reduced caspase-3/7 activity relative to untreated AMD cybrids (AMD HNF_14_: 0.772 ± 0.033; *p* < 0.001). Co-treatment with HNF_14_ and Aβ_1–42_ significantly attenuated Aβ_1–42_-induced caspase-3/7 activation compared with Aβ_1–42_ treatment alone (AMD Amyloid-β_1–42_: 1.150 ± 0.032; AMD HNF_14_ + Amyloid-β_1–42_: 0.898 ± 0.081; *p* < 0.001). In contrast, treatment with the scrambled control peptide (Aβ_42–1_-SC) did not significantly alter caspase-3/7 activity relative to untreated AMD cybrids (AMD SC: 0.978 ± 0.046; ns), and co-treatment with HNF_14_ and Aβ_42–1_-SC did not differ from untreated controls (AMD HNF_14_ + SC: 0.934 ± 0.068; ns).

## 4. Discussion

In this study, we demonstrate that HNF_14_, a 14-amino acid fragment derived from the Humanin peptide, exerts robust cytoprotective effects in AMD transmitochondrial cybrid cells. HNF_14_ treatment selectively improved cellular metabolic activity, reduced oxidative stress, suppressed cytotoxicity and apoptotic signaling, and modulated inflammatory and angiogenic markers in AMD cybrids, while exerting minimal effects in normal cybrids. These findings extend the functional relevance of mitochondrial-derived peptides in AMD and identify HNF_14_ as a disease-selective modulator of mitochondrial-associated cellular stress pathways.

Mitochondrial dysfunction is a defining feature of AMD and is strongly linked to RPE degeneration. Prior studies using AMD cybrids have shown that mitochondria derived from AMD patients are intrinsically damaged and capable of driving cellular stress responses independent of nuclear genetic background [9,13]. These stress responses include elevated reactive oxygen species [22], mitochondrial DNA damage, impaired metabolic activity, and activation of apoptotic and inflammatory pathways [4,5,6,9,14]. The present findings demonstrate that HNF_14_ significantly attenuates these pathological features, supporting the concept that mitochondrial-targeted modulation can influence disease-associated cellular phenotypes in this model.

Oxidative stress plays a central role in AMD pathogenesis, and HNF_14_ markedly reduced both intracellular reactive oxygen species and mitochondrial superoxide levels in AMD cybrids. This reduction was accompanied by upregulation of mitochondrial antioxidant defenses suggesting that HNF_14_ may augment endogenous protective mechanisms rather than acting solely as a passive antioxidant. These findings are consistent with prior studies showing that Humanin and HNG mitigate oxidative stress in AMD cybrids and reinforce the notion that Humanin-derived peptides modulate mitochondrial redox homeostasis [9,10,11,23].

Apoptosis is a key driver of RPE cell loss in AMD. AMD cybrids exhibited elevated markers of cytotoxicity and apoptosis, including increased Caspase-3/7 activation, consistent with previous reports [9,11,24]. Extracellular Annexin V levels were assessed as a complementary, population-level marker of apoptosis and were interpreted in conjunction with caspase-3/7 activity and cleaved caspase-3 protein measurements. HNF_14_ treatment significantly suppressed these apoptotic indicators, suggesting stabilization of mitochondrial integrity and downstream survival signaling. Notably, these anti-apoptotic effects were selective for AMD cybrids, reinforcing the disease-specific action of HNF_14_. This selectivity mirrors prior observations with HNG and supports the concept that mitochondrial-derived peptides preferentially engage stress-activated pathways present in diseased mitochondria [9,10,11,12,25].

Inflammatory signaling is increasingly recognized as a contributor to AMD progression, particularly through interactions between mitochondrial dysfunction and innate immune activation. Prior studies have demonstrated that Humanin and HNG reduce inflammatory marker expression in AMD cybrids and patient plasma [11]. In the current study, HNF_14_ reduced expression of inflammatory mediators such as IL-1β, suggesting that shorter Humanin fragments may also modulate mitochondria-driven inflammatory pathways. This observation may be relevant to AMD pathophysiology, where chronic inflammation contributes to progressive retinal degeneration [26].

Another clinically relevant finding was the reduction in VEGF-A protein levels in AMD cybrids treated with HNF_14_. Aberrant VEGF signaling is central to neovascular AMD, and mitochondrial dysfunction has been linked to dysregulated angiogenic factor expression [27]. Previous studies have shown that HNG normalizes angiogenesis-related proteins in AMD cybrids [12]. The present results indicate that HNF_14_ similarly suppresses VEGF-A expression, suggesting potential relevance to pathways implicated in both neovascular and non-neovascular forms of AMD.

A particularly compelling aspect of this study is the demonstration that HNF_14_ protects AMD cybrids from amyloid-β_1–42_-induced apoptosis. Amyloid-β accumulation has been implicated in drusen formation and RPE toxicity in AMD [28,29,30]. Prior work established that HNG protects AMD cybrids from amyloid-β-mediated mitochondrial and cellular damage [9]. The ability of HNF_14_ to confer similar protection indicates that essential anti-amyloidogenic and anti-apoptotic properties are retained within this shorter peptide fragment.

Collectively, these findings suggest that HNF_14_ preserves key functional domains of Humanin that are sufficient to rescue mitochondrial and cellular dysfunction in AMD. The use of a shorter fragment peptide may offer practical advantages for experimental and translational investigation while maintaining disease-selective activity. Importantly, the selective action of HNF_14_ in AMD cybrids minimizes concerns regarding off-target effects in healthy cells.

Several limitations should be acknowledged. This study was conducted using an in vitro cybrid model, and future studies will be required to evaluate the in vivo efficacy, delivery, and safety of HNF_14_. Additionally, while mitochondrial stress markers were significantly improved, further investigation into bioenergetic parameters such as mitochondrial respiration and ATP production would provide additional mechanistic insight.

In conclusion, this study demonstrates that HNF_14_, a Humanin-derived fragment peptide, mitigates mitochondrial and cellular stress responses in AMD transmitochondrial cybrid cells. HNF_14_ treatment was associated with reduced oxidative stress, apoptotic signaling, inflammatory markers, angiogenic factor expression, and amyloid-β_1–42_-induced cytotoxicity in a disease-specific mitochondrial context. These findings extend prior work on mitochondrial-derived peptides by showing that a shorter Humanin fragment retains cytoprotective activity in AMD cybrids. Collectively, this study supports further investigation of mitochondrial-derived peptides as modulators of mitochondrial dysfunction relevant to AMD pathophysiology.

## Data Availability

The raw data supporting the conclusions of this article will be made available by the authors on reasonable request, in accordance with institutional and ethical guidelines.
